# Advances in the mechanisms of electroacupuncture in the treatment of cerebral ischemia–reperfusion injury

**DOI:** 10.3389/fnins.2026.1806298

**Published:** 2026-04-13

**Authors:** Hong Yang, Zhan-Ze Ma, Jun Liu

**Affiliations:** 1 Third Department of Encephalopathy Rehabilitation, Affiliated Hospital of Liaoning University of Traditional Chinese Medicine, Shenyang, China; 2 Department of Integrated Traditional Chinese and Western Medicine, Liaoning University of Traditional Chinese Medicine Xinglin College, Shenyang, China

**Keywords:** blood–brain barrier integrity, cerebral ischemia–reperfusion injury, electroacupuncture, mechanisms, neuroprotection

## Abstract

Cerebral ischemia–reperfusion injury (CIRI) is a critical pathological process that adversely affects neurological recovery and prognosis after reperfusion therapies for ischemic stroke, and effective targeted interventions remain limited. Accumulating experimental and clinical evidence indicates that electroacupuncture (EA), as a traditional Chinese medicine–based intervention with controllable stimulation parameters, can attenuate CIRI and improve neurological outcomes to a certain extent. Current studies suggest that the neuroprotective effects of EA involve integrated modulation of multiple pathological processes, including neuronal apoptosis, inflammatory responses, oxidative stress, blood–brain barrier disruption, angiogenesis, and synaptic plasticity, exhibiting characteristics of multi-target, multi-pathway, and dynamic regulation. Meanwhile, EA stimulation parameters, acupoint selection, and intervention timing play important roles in determining therapeutic efficacy. Based on an overview of the major pathological mechanisms of CIRI, this review systematically summarizes the key molecular pathways and parameter-related features underlying EA intervention, aiming to provide references for mechanistic integration and clinical application of EA in CIRI.

## 1 Introduction

Ischemic stroke is a common cerebrovascular disease that poses a serious threat to human health worldwide (Zhang et al., 2024). In recent years, with the rapid development of intravenous thrombolysis and endovascular thrombectomy, an increasing number of patients can achieve vascular recanalization during the acute phase, leading to a marked improvement in survival rates (Stoll et al., 2024). However, extensive clinical and experimental studies have demonstrated that restoration of blood flow does not necessarily indicate termination of brain injury. On the contrary, reperfusion itself may induce or exacerbate cerebral damage, a phenomenon known as cerebral ischemia–reperfusion injury (CIRI) (Meng et al., 2025).

CIRI occurs with a relatively high incidence in clinical settings and represents one of the major causes of poor neurological recovery, cognitive impairment, and long-term disability after ischemic stroke (Yang et al., 2025). Its pathological processes are highly complex, involving neuronal death, amplification of inflammatory responses, exacerbation of oxidative stress, and formation of cerebral edema, among other mechanisms (Jurcau and Ardelean, 2021). Notably, even after successful thrombolysis or thrombectomy, some patients continue to experience neurological deterioration, suggesting that restoration of cerebral perfusion alone is insufficient to fully halt the progression of injury (Xu et al., 2025). Therefore, effective intervention strategies targeting CIRI have become an urgent clinical challenge in contemporary stroke management.

Electroacupuncture (EA) is a therapeutic modality derived from traditional manual acupuncture with the incorporation of modern electrical stimulation, characterized by controllable parameters, sustained stimulation, and relatively good reproducibility of therapeutic effects (Yang et al., 2026). Compared with manual acupuncture, EA is more suitable for mechanistic studies and standardized clinical application. A growing body of evidence indicates that EA exerts beneficial effects in improving cerebral blood flow, protecting neurons, suppressing inflammatory responses, and promoting functional recovery (Hou et al., 2025). Through stable and continuous electrical stimulation applied to specific acupoints, EA is thought to modulate systemic qi and blood circulation while improving local cerebral functional states, providing a theoretical basis within traditional Chinese medicine for the intervention of CIRI. Accordingly, systematic elucidation of the mechanisms underlying EA treatment for CIRI is of considerable significance for advancing integrative medicine research and clinical translation.

In summary, this review systematically examines experimental studies on CIRI published in the past 5 years. A comprehensive literature search was conducted in databases including PubMed, Web of Science, and China National Knowledge Infrastructure (CNKI) using keywords such as cerebral ischemia–reperfusion injury, electroacupuncture, neuroprotection, apoptosis, autophagy, and oxidative stress, combined with Boolean operators (AND, OR). Studies were included based on predefined criteria focusing on original research investigating the mechanisms of EA intervention in CIRI models. This review aims to summarize the key mechanisms by which EA intervenes in CIRI from multiple perspectives, thereby providing a reference for the standardized application of EA in preclinical settings and for guiding further mechanistic investigations.

## 2 Core pathophysiological mechanisms of CIRI

Cerebral ischemia–reperfusion injury is not caused by a single mechanism, but rather results from the interaction and mutual amplification of multiple pathological processes across temporal and spatial dimensions.

### 2.1 Excessive neuronal apoptosis as a central mechanism of neural injury

Under ischemic and hypoxic conditions, cerebral energy metabolism is disrupted, mitochondrial function is impaired, and adenosine triphosphate (ATP) production is reduced, leading to intracellular calcium homeostasis imbalance (Zhang et al., 2024). Upon reperfusion, increased mitochondrial membrane permeability promotes the release of pro-apoptotic factors and activates intrinsic apoptotic pathways (Fang et al., 2025). Imbalance of the B-cell lymphoma 2 (Bcl-2)/Bcl-2-associated X protein (Bax) ratio and the sequential activation of Caspase-9 and Caspase-3 represent key molecular events in this process (Wang et al., 2025). In addition, death receptor–mediated extrinsic apoptotic pathways are also involved, as activation of Fas cell surface death receptor/Fas ligand (Fas/FasL) signaling can further amplify neuronal programmed cell death (Yang et al., 2025). Extensive neuronal apoptosis within ischemia–reperfusion regions constitutes a core pathological basis for structural disruption and functional impairment of brain tissue (Hu et al., 2017; Yan et al., 2021).

### 2.2 Cascade amplification of inflammatory responses aggravates secondary brain injury

Cerebral ischemia–reperfusion rapidly activates inflammation-related signaling pathways. As a key transcription factor, nuclear factor of Kappa light polypeptide gene enhancer in B-cells (NF-κB) undergoes nuclear translocation during the early reperfusion phase, inducing the excessive release of pro-inflammatory cytokines such as tumor necrosis factor alpha (TNF-α), interleukin 1 beta (IL-1β), and interleukin 6 (IL-6) (Jover-Mengual et al., 2021). Meanwhile, microglia polarize from a resting state toward the pro-inflammatory M1 phenotype, astrocytes become excessively activated, and peripheral immune cells infiltrate the ischemic region, collectively forming a sustained inflammatory microenvironment (Liu et al., 2025). Recent studies have demonstrated that NLR family pyrin domain containing 3 inflammasome–mediated pyroptosis plays an important role in cerebral ischemia–reperfusion injury (Xu et al., 2025). Activation of this pathway leads to Caspase-1 cleavage and maturation of inflammatory cytokines, further amplifying inflammatory responses and acting as a critical driver of secondary injury (Jover-Mengual et al., 2021).

### 2.3 Oxidative stress imbalance and excessive accumulation of reactive oxygen species

During reperfusion, large amounts of reactive oxygen species (ROS) are generated, while antioxidant defense systems are relatively insufficient, resulting in an imbalance between oxidation and antioxidation (Sun et al., 2018). Excessive ROS can attack membrane lipids, induce lipid peroxidation, and generate toxic products such as malondialdehyde, while also damaging proteins and DNA, thereby aggravating neuronal injury (Bi et al., 2017). Oxidative stress not only directly causes cellular damage but also activates inflammasomes and cell death pathways, forming a vicious cycle with inflammatory responses and apoptosis (Sun et al., 2018).

### 2.4 Disruption of blood–brain barrier integrity and vasogenic cerebral edema

Cerebral ischemia–reperfusion leads to degradation of tight junction proteins in the blood–brain barrier (BBB), including tight junction protein 1, Occludin, and Claudin-5, resulting in increased vascular permeability (Liao et al., 2021). Concurrently, abnormal upregulation of the water channel protein aquaporin-4 (AQP4) promotes excessive water extravasation into brain tissue, leading to vasogenic cerebral edema, increased intracranial pressure, and further exacerbation of brain injury (Bi et al., 2017).

### 2.5 Insufficient angiogenesis and impaired restoration of cerebral perfusion

Damage to vascular endothelial cells in ischemic regions and insufficient expression of angiogenic factors disrupt microvascular structure, leading to uneven blood flow distribution after reperfusion and limited restoration of local perfusion (Ruan et al., 2025; Zhang et al., 2017). Insufficient angiogenic capacity not only restricts oxygen and nutrient supply but also impairs neural repair and functional reconstruction (Mei et al., 2020).

### 2.6 Impaired synaptic plasticity and obstructed neural circuit reconstruction

Cerebral ischemia–reperfusion injury reduces the secretion of neurotrophic factors and downregulates the expression of synapse-related proteins, resulting in alterations in synaptic number and structure (Yang et al., 2021; Gudasheva et al., 2019). Impairment of synaptic plasticity hinders neural circuit reconstruction and constitutes an important structural basis for deficits in learning, memory, and motor function recovery (Dietz et al., 2018; Han et al., 2023).

### 2.7 Other key pathological processes

In addition to the mechanisms described above, cerebral ischemia–reperfusion injury also involves calcium overload, dysregulation of autophagy, ferroptosis, endoplasmic reticulum stress, and inflammation mediated by the gut–brain axis (Tang et al., 2025).

**Figure 1:**
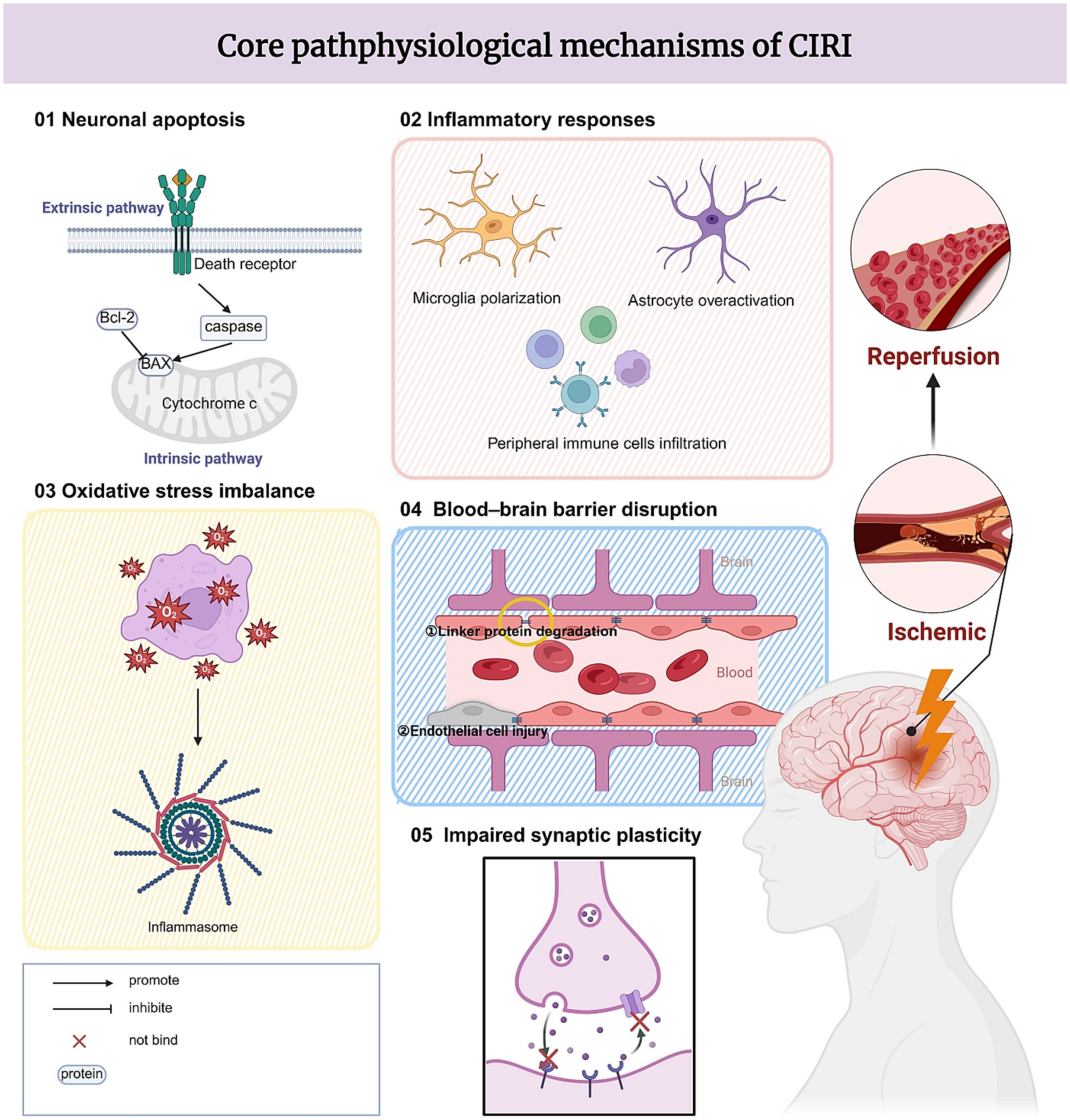
Core pathological mechanism of CIRI.

In this section, to facilitate a systematic discussion, we have categorized the complex pathological processes of CIRI into five core components: neuronal apoptosis, inflammatory response, oxidative stress imbalance, blood–brain barrier disruption, and impaired synaptic plasticity (Figure 1). The key molecular events underlying each component have been analyzed individually. Under actual pathological conditions, however, these five components engage in extensive crosstalk and are far from being isolated linear events.

The burst of ROS during early reperfusion induces oxidative stress imbalance. These excessive ROS not only serve as direct cytotoxic molecules but also function as a hub connecting the other four pathological components. On one hand, ROS trigger the mitochondrial apoptotic pathway, driving excessive neuronal apoptosis. On the other hand, they activate NF-κB and the NLRP3 inflammasome, initiating the cascade amplification of inflammatory responses (Guo et al., 2024). Subsequently, activated M1 microglia release pro-inflammatory cytokines (TNF-α, IL-1β), which attack vascular endothelial cells and degrade tight junction proteins, resulting in blood–brain barrier disruption. Following barrier integrity loss, peripheral inflammatory cell infiltration further exacerbates central inflammation, forming a vicious cycle. Ultimately, the persistent oxidative stress and inflammatory microenvironment inhibit neurotrophic factor secretion, leading to impaired synaptic plasticity and manifesting as neurological dysfunction (Versele et al., 2022).

Collectively, these five core mechanisms interact with and reinforce one another, forming a complex pathological network that is further intertwined with other processes such as calcium overload, autophagy dysregulation, ferroptosis, endoplasmic reticulum stress, and gut-brain axis-mediated inflammation, thereby increasing the difficulty of intervention.

This complex pathological network inherently limits the efficacy of single-target interventions, raising the critical question of whether a multi-target approach such as electroacupuncture may offer advantages. This question guides our subsequent discussion on the mechanisms of EA in treating CIRI.

This figure illustrates the main pathophysiological mechanisms involved in CIRI, including neuronal apoptosis, inflammatory responses, oxidative stress imbalance, blood–brain barrier disruption, and impaired synaptic plasticity. Although reperfusion restores blood flow after ischemia, it can also trigger excessive production of reactive oxygen species and activate inflammatory responses. These processes contribute to neuronal death, damage to the blood–brain barrier, and alterations in synaptic function, ultimately leading to neurological dysfunction.

## 3 Core mechanisms of EA in the treatment of CIRI

### 3.1 Regulation of neuronal apoptosis and related programmed cell death pathways

Neuronal apoptosis is one of the earliest-initiated forms of cell death in cerebral ischemia–reperfusion injury and persists throughout disease progression, with its extent directly determining the survival outcome of neurons in the ischemic penumbra (Gong et al., 2017). Ischemia, hypoxia, and reperfusion stress induce neuronal programmed cell death through multiple mechanisms, including mitochondrial dysfunction, death receptor activation, and endoplasmic reticulum stress (Wang et al., 2022). Electroacupuncture modulates apoptosis-related signaling pathways at multiple levels, thereby suppressing excessive neuronal apoptosis.

#### 3.1.1 Activation of the phosphoinositide 3-kinase/serine–threonine kinase (PI3K/AKT) and downstream anti-apoptotic signaling axis

The PI3K/Akt signaling pathway is a critical anti-apoptotic pathway that maintains neuronal survival and confers resistance to ischemic stress (Lan et al., 2013). Studies have shown (Huang et al., 2024; Su et al., 2023; Wang, 2022; Quan et al., 2021) that electroacupuncture stimulation at acupoints such as Baihui, Shenting, Shuigou, Zusanli, and Neiguan significantly upregulates the expression of PI3K, Akt, and its phosphorylated form p-Akt in brain tissue within ischemia–reperfusion regions. Activation of Akt suppresses the activity of multiple pro-apoptotic signaling molecules, thereby interrupting apoptotic cascade reactions (Kennedy et al., 1999).

At the molecular level, Akt can inhibit pro-apoptotic proteins such as Bcl-2 associated agonist of cell death (BAD) and Glycogen Synthase Kinase 3 Beta (GSK-3β), reduce the cleavage of Caspase-9 and Caspase-3, and consequently attenuate neuronal programmed cell death (Huang et al., 2024; Quan et al., 2021; Hao et al., 2025; Zhu, 2024). In addition, several studies suggest that electroacupuncture may regulate cellular energy metabolism and protein synthesis via the PI3K/AKT/mechanistic target of rapamycin kinase (mTOR) signaling axis, enhancing neuronal tolerance to ischemia–reperfusion stress (Huang et al., 2024; Su et al., 2023; Zhu, 2024).

Moreover, the PI3K/Akt pathway is closely associated with stabilization of hypoxia-inducible factor-1α (HIF-1α) expression in ischemic regions. Evidence indicates that electroacupuncture-induced activation of PI3K/Akt signaling upregulates HIF-1α levels, thereby improving the metabolic microenvironment of ischemic tissue and indirectly supporting neuronal survival and subsequent vascular repair (Quan et al., 2021; Liao et al., 2025; Yuan et al., 2025), highlighting the pivotal role of this pathway across multiple pathological processes.

#### 3.1.2 Regulation of the Bcl-2/Bax ratio to maintain mitochondrial homeostasis and block intrinsic apoptotic pathways

Mitochondria serve as central organelles for integration of apoptotic signals in cerebral ischemia–reperfusion injury (Zhang et al., 2025). Members of the Bcl-2 family regulate mitochondrial outer membrane permeability and play a decisive role in determining cell survival or death (King et al., 2023; Qian et al., 2022). Extensive studies have demonstrated that electroacupuncture markedly increases the expression of the anti-apoptotic protein Bcl-2 while decreasing the expression of the pro-apoptotic protein Bax, thereby elevating the Bcl-2/Bax ratio and maintaining mitochondrial membrane stability (Chen et al., 2026; Ding, 2023; Ding et al., 2022; Zhong et al., 2022).

By stabilizing mitochondrial outer membrane integrity, electroacupuncture suppresses the release of pro-apoptotic factors such as cytochrome c and prevents activation of the Caspase cascade (Chen et al., 2026; Ding, 2023; Zhong et al., 2022). Furthermore, several studies (Chen et al., 2026; Ding, 2023; Ding et al., 2022; Liang et al., 2023) have reported that electroacupuncture can inhibit excessive mitochondrial fission and promote mitochondrial fusion, thereby improving mitochondrial dynamics and providing sustained energy support for neurons, ultimately enhancing resistance to apoptotic injury.

#### 3.1.3 Intervention in endoplasmic reticulum stress and extrinsic apoptotic pathways

In addition to mitochondria-mediated intrinsic apoptosis, endoplasmic reticulum stress and death receptor–mediated extrinsic apoptosis also contribute to the development of cerebral ischemia–reperfusion injury. During reperfusion, disturbances in calcium homeostasis and protein folding induce endoplasmic reticulum stress responses, activating signaling molecules such as inositol-requiring enzyme 1 alpha (IRE1α), TNF receptor associated factor 2 (TRAF2), and apoptosis signal-regulating kinase 1 (ASK1), and subsequently triggering Caspase-12–mediated apoptotic pathways (Kang et al., 2024; Zheng et al., 2025; Zhu and Zhou, 2021).

Studies have shown that electroacupuncture attenuates endoplasmic reticulum stress by regulating the IRE1α/TRAF2/ASK1/Caspase-12 signaling axis, thereby inhibiting stress-induced neuronal apoptosis (Nie et al., 2023; Fu, 2023; Fu et al., 2023). In addition, some evidence suggests that electroacupuncture may activate the IRE1α/receptor for activated C kinase 1 (RACK1) molecular switch to balance endoplasmic reticulum stress and cell survival signaling (Fu, 2023). With respect to extrinsic apoptosis, electroacupuncture also suppresses Fas/FasL-mediated death receptor signaling, reducing the contribution of extrinsic apoptotic pathways to neuronal death (Nie et al., 2023; Fu et al., 2023). Together, these anti-apoptotic effects represent a fundamental neuroprotective mechanism of electroacupuncture in CIRI.

### 3.2 Suppression of inflammatory responses and pyroptotic networks to block secondary injury amplification

Inflammatory responses and inflammation-related pyroptotic processes represent one of the most critical secondary injury amplification mechanisms in cerebral ischemia–reperfusion injury (Zheng et al., 2022). Unlike apoptosis alone, inflammation-associated cell death is typically accompanied by massive release of inflammatory mediators, which establishes a persistent inflammatory microenvironment within and around ischemic regions, thereby exacerbating neuronal injury (Song et al., 2022; Rao et al., 2022). Electroacupuncture inhibits inflammatory responses and pyroptosis through multi-target modulation of inflammatory signaling networks.

#### 3.2.1 Inhibition of the NF-κB inflammatory signaling pathway and its endogenous regulatory network

NF-κB is a central transcription factor responsible for initiation and amplification of inflammatory responses in cerebral ischemia–reperfusion injury. Following reperfusion, the NF-κB p65 subunit rapidly translocates into the nucleus, inducing excessive expression of pro-inflammatory cytokines such as TNF-α, IL-1β, and IL-6, thereby triggering inflammatory cascades (Jover-Mengual et al., 2021; Wu et al., 2021). Multiple studies have demonstrated that electroacupuncture intervention markedly suppresses NF-κB activation and reduces pro-inflammatory cytokine levels, thereby exerting early inhibitory effects on inflammatory responses (Bai et al., 2024; Luo et al., 2024a; Zhuang et al., 2022).

At a more refined regulatory level, electroacupuncture does not simply inhibit NF-κB signaling but rather achieves precise modulation by enhancing endogenous negative regulatory networks. Specifically, electroacupuncture has been shown to upregulate the expression of NF-κB inhibitory proteins such as A20-binding inhibitor of NF-κB activation 1 (ABIN1), TNF alpha induced protein 3 (A20), and Cezanne, thereby preventing sustained activation of NF-κB signaling (Zhou, 2021; Lu et al., 2021). Silencing of ABIN1 partially attenuates the anti-inflammatory effects of electroacupuncture (Zhou, 2021), suggesting that the ABIN1/A20 axis plays a critical role in electroacupuncture-mediated suppression of NF-κB signaling.

#### 3.2.2 Parallel regulation of NLRP3, NLRP1, and absent in melanoma 2 (AIM2) inflammasome axes

Recent studies have established that inflammasome-mediated pyroptosis occupies a central position in cerebral ischemia–reperfusion injury. Activation of the NLRP3 inflammasome induces Caspase-1 cleavage, promotes Gasdermin D (GSDMD) pore formation, and facilitates maturation and release of IL-1β and interleukin 18 (IL-18), thereby amplifying inflammatory responses (Groslambert and Py, 2018; Yu et al., 2021). Numerous studies consistently demonstrate that electroacupuncture significantly downregulates the expression of NLRP3, apoptosis-associated speck-like protein containing a CARD (ASC), Caspase-1, and GSDMD, effectively suppressing pyroptotic cell death (Hao et al., 2025; Yu et al., 2026; Chen et al., 2024; You et al., 2023; Wang and Wang, 2022; Liu Z. Q. et al., 2022; Luo et al., 2022).

In addition to NLRP3, the NLRP1 and AIM2 inflammasomes also contribute to inflammatory amplification in cerebral ischemia–reperfusion injury. Related studies (Zheng et al., 2025; Chen et al., 2023) indicate that electroacupuncture combined with other interventions can inhibit AIM2 inflammasome-mediated pyroptosis, further expanding the regulatory scope of electroacupuncture on inflammasome networks. Collectively, these findings suggest that electroacupuncture exerts parallel regulation over multiple inflammasome axes rather than targeting a single inflammasome pathway.

#### 3.2.3 Regulation of the thioredoxin-interacting protein (TXNIP)/ROS/NLRP3 axis linking oxidative stress and pyroptosis

TXNIP is considered a critical molecular hub linking oxidative stress to inflammasome activation (Fei et al., 2023). Under reperfusion conditions, excessive reactive oxygen species (ROS) promote dissociation of TXNIP from the thioredoxin complex, allowing TXNIP to directly bind NLRP3 and trigger inflammasome activation (Jiang et al., 2022; Choi and Park, 2023). Studies have shown that electroacupuncture pretreatment or post-reperfusion intervention significantly downregulates TXNIP expression and suppresses activation of the ROS/TXNIP/NLRP3 signaling axis (Jiang et al., 2025; Wu et al., 2025; Pang, 2024). Through this mechanism, electroacupuncture alleviates oxidative stress and inhibits inflammasome-mediated pyroptosis, thereby coordinating antioxidative and anti-inflammatory effects.

#### 3.2.4 Modulation of cyclic GMP-AMP synthase-stimulator of interferon genes (cGAS–STING) and other inflammatory signaling axes

Beyond classical inflammasome pathways, the cGAS–STING signaling pathway has attracted increasing attention for its role in cerebral ischemia–reperfusion injury. This pathway senses damage-associated DNA signals and induces inflammatory cytokine expression, thereby amplifying inflammatory responses (Wang et al., 2024). Studies indicate that electroacupuncture suppresses expression of cGAS–STING and its downstream signaling components, resulting in attenuation of inflammatory responses and associated cell death (Wang et al., 2025; Zhang et al., 2025). In addition, electroacupuncture has been shown to inhibit other inflammation-related pathways, including Toll-like receptor 4 (TLR4)/myeloid differentiation primary response 88 (MyD88)/NF-κB and interleukin 17A (IL-17A) signaling (Bai et al., 2024; Luo et al., 2024a; Zhao et al., 2025), further broadening its anti-inflammatory and anti-pyroptotic regulatory spectrum.

### 3.3 Activation of antioxidant stress pathways and suppression of ferroptosis to alleviate reperfusion-induced oxidative injury

Oxidative stress represents one of the earliest and most critical events in the development of cerebral ischemia–reperfusion injury. During reperfusion, excessive ROS generation overwhelms endogenous antioxidant defenses, leading to enhanced lipid peroxidation and forming a vicious cycle with inflammatory responses and cell death processes (Du et al., 2025; Granger and Kvietys, 2015). Accumulating evidence demonstrates that electroacupuncture alleviates oxidative stress–related injury through multi-level regulation of antioxidant signaling pathways.

#### 3.3.1 Activation of the nuclear factor erythroid 2-related factor (NRF2)/heme oxygenase-1 (HO-1) antioxidant signaling network

Nrf2 is a central transcription factor governing intracellular antioxidant responses. Studies have shown that electroacupuncture stimulation at acupoints such as Baihui, Shenting, and Zusanli significantly promotes nuclear translocation of NRF2 and upregulates downstream antioxidant genes including HO-1 and NAD(P)H quinone dehydrogenase 1 (NQO1) (Zhu, 2024; Shi et al., 2026; Zhang et al., 2025; Zhao, 2025; Zhang et al., 2023). Concurrently, electroacupuncture increases superoxide dismutase (SOD) and glutathione peroxidase (GSH-Px) activities while reducing levels of lipid peroxidation products such as Malondialdehyde (MDA) in brain tissue (Zhang et al., 2023; Yan et al., 2022), thereby effectively scavenging excessive ROS generated during reperfusion.

#### 3.3.2 Regulation of iron metabolism dysregulation and ferroptosis-related pathways

Emerging evidence indicates that ferroptosis plays an important role in cerebral ischemia–reperfusion injury, closely associated with iron metabolism dysregulation and lipid peroxidation. Studies demonstrate that electroacupuncture upregulates glutathione (GSH) and glutathione peroxidase 4 (GPX4) expression while downregulating key ferroptosis-related proteins such as acyl-CoA synthetase long chain family member 4 (ACSL4) and transferrin receptor (TFRC), thereby suppressing lipid peroxidation and ferroptotic cell death (Zhu, 2024; Zhang et al., 2025; Wu et al., 2023).

### 3.4 Preservation of blood–brain barrier integrity and neurovascular unit homeostasis

Disruption of the blood–brain barrier (BBB) and dysfunction of the neurovascular unit constitute important structural bases of cerebral ischemia–reperfusion injury (Blase et al., 2025). During reperfusion, endothelial injury, degradation of tight junction proteins, and abnormal expression of water channel proteins collectively increase BBB permeability and promote cerebral edema formation (Chen et al., 2012; Kim et al., 2020). Studies indicate that electroacupuncture stimulation at acupoints such as Baihui, Shuigou, and Shenting significantly upregulates tight junction proteins including ZO-1 and Occludin while suppressing abnormal elevation of aquaporin 4 (AQP4), thereby preserving BBB integrity and alleviating vasogenic cerebral edema (Liao et al., 2025; Lv et al., 2026; Gong et al., 2023; Deng et al., 2022).

Furthermore, epigenetic studies suggest that electroacupuncture participates in BBB protection via regulation of m6A methylation. Specifically, electroacupuncture downregulates METTL3 and methyltransferase 3, N6-adenosine-methyltransferase complex catalytic subunit (METTL3)-mediated m6A modification of long non-coding RNA H19 (lncRNA H19), suppressing activation of the sphingosine-1-phosphate receptor 2 (S1PR2)/TLR4/NLRP3 signaling axis, thereby reducing inflammatory responses and maintaining BBB integrity (Zhang, 2025).

### 3.5 Promotion of angiogenesis and microcirculatory reconstruction to improve cerebral perfusion

Angiogenesis constitutes a critical foundation for tissue repair and functional recovery following cerebral ischemia–reperfusion injury. Evidence indicates that electroacupuncture promotes angiogenesis and microcirculatory reconstruction through multiple signaling pathways.

Studies demonstrate that electroacupuncture significantly upregulates expression of hypoxia inducible factor 1 subunit alpha (HIF-1α), vascular endothelial growth factor (VEGF), and related receptors, promoting endothelial cell proliferation, migration, and tube formation, thereby increasing microvascular density in ischemic regions (Liao et al., 2025; Yuan et al., 2025; Hua et al., 2025; Yang et al., 2025; Tan et al., 2023). Recent findings further reveal that electroacupuncture enhances the generation of exosomal microRNA-210 (miR-210) and activates the HIF-1α/VEGF/Notch signaling pathway, thereby augmenting angiogenic capacity (Yuan et al., 2025). In addition, electroacupuncture pretreatment increases circulating endothelial progenitor cell levels and improves cerebral blood flow (Zhang, 2024), suggesting involvement of peripheral reparative cell mobilization in cerebrovascular reconstruction.

### 3.6 Regulation of synaptic plasticity and neural network remodeling to facilitate functional recovery

Impairment of synaptic plasticity represents a key structural basis for deficits in learning, memory, and motor function following cerebral ischemia–reperfusion injury. Extensive studies demonstrate that electroacupuncture regulates synaptic structure and function through multiple pathways, thereby promoting neural network reconstruction. Specifically, electroacupuncture upregulates expression of brain derived neurotrophic factor (BDNF), tropomyosin-related kinase B (TRKB), and downstream cAMP response element-binding protein (CREB), promoting synthesis of synaptic proteins such as postsynaptic density protein 95 (PSD-95) and Synapsin-1, ultimately improving synaptic number and morphology (Lin et al., 2024; Cheng J. et al., 2024; Yuan et al., 2023). This process is considered a fundamental molecular basis underlying electroacupuncture-mediated improvement of learning and memory function.

Moreover, electroacupuncture modulates glutamatergic and GABAergic systems by suppressing excessive activation of N-methyl-D-aspartate receptor subunit 2B (NMDAR2B), neuronal Nitric Oxide Synthase (NNOS), and alcium/calmodulin-dependent protein kinase II (CAMK-II) while upregulating glutamate receptor 2 (GluR-2) and gamma-aminobutyric acid (GABA) receptor subunits, thereby attenuating excitotoxic injury and restoring synaptic transmission homeostasis (Li, 2022; Yang et al., 2024; Han and Zhang, 2022).

### 3.7 Integrated regulation of mitochondrial homeostasis, autophagy, and the gut–brain axis for systemic neuroprotection

Beyond the mechanisms described above, electroacupuncture exerts systemic neuroprotective effects by regulating mitochondrial homeostasis, autophagy, and gut–brain axis function. Studies indicate that electroacupuncture modulates mitochondrial fusion-related proteins such as optic atrophy protein 1 (OPA1) and mitofusin 2 (MFN2), suppresses excessive mitochondrial fission, reduces release of pro-apoptotic factors, and promotes mitochondrial biogenesis via the peroxisome proliferator-activated receptor gamma coactivator 1-alpha (PGC-1α)/nuclear respiratory factor 1 (NRF1)/mitochondrial transcription factor A (TFAM) pathway, thereby maintaining mitochondrial functional stability (Wang, 2022; Chen et al., 2026; Ding, 2023; Ding et al., 2022; Zhong et al., 2022; Liang et al., 2023; Yuan, 2024).

With respect to autophagy regulation, electroacupuncture exhibits distinct stage-dependent bidirectional effects. During the early reperfusion phase, electroacupuncture suppresses excessive autophagy through pathways such as AMPK/mTOR/ULK1, thereby reducing neuronal injury (Luo et al., 2026; Su et al., 2025; Luo et al., 2024b). During the recovery phase, electroacupuncture promotes mitophagy via the Unc-51 like autophagy activating kinase 1 (PINK1)/Parkin and Bcl-2 interacting protein 3 like (BNIP3L) pathways, facilitating clearance of damaged mitochondria and improving mitochondrial quality control (Ding, 2023; Zhong et al., 2022; Xu et al., 2024; Liu D. N. et al., 2022; Xu et al., 2021; Feng, 2021).

**Figure 2:**
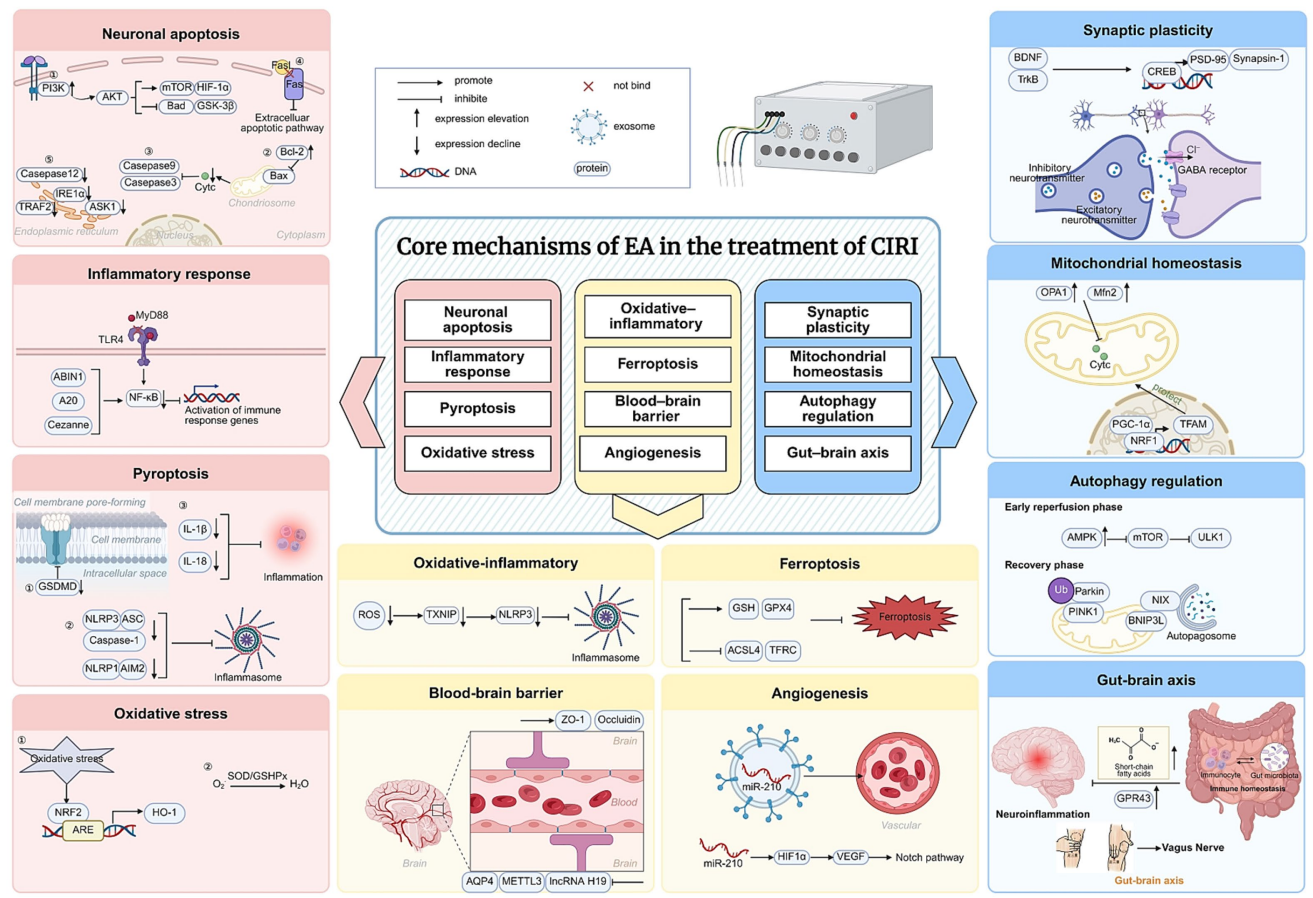
Schematic of the core mechanisms of electroacupuncture (EA) in cerebral ischemia reperfusion injury (CIRI).

In addition, recent studies demonstrate that electroacupuncture modulates gut microbiota composition, increases levels of short-chain fatty acids, activates G protein-coupled receptor 43 (GPR43) signaling, and regulates regulatory T cell (Treg)/gamma delta T cell (γδT) cell immune balance, thereby attenuating gut–brain inflammatory responses (Pang, 2024; Yan et al., 2026a; Yan et al., 2026b; Zhao, 2025; Zhang et al., 2021; Wang, 2021). Low-intensity electroacupuncture at Zusanli further improves intestinal barrier function via vagus nerve–dependent mechanisms (Zhang et al., 2021; Wang, 2021), highlighting the systemic regulatory characteristics of electroacupuncture in cerebral ischemia–reperfusion injury (Figure 2 and Table 1).

**Table 1:** Mechanisms of EA in intervening CIRI.

Pathological process	Primary cell/structure	Key molecule or signaling pathway	EA action direction	Functional outcome
Neuronal apoptosis	Neurons	PI3K/AKT/mTOR	Activation↑	Inhibit apoptosis and promote neuronal survival
		Bcl-2↑/Bax↓	Increased ratio	Stabilize the outer mitochondrial membrane and block intrinsic apoptosis
		Caspase-9/Caspase-3	Inhibition↓	Reduce programmed cell death
		Fas/FasL	Inhibition↓	Inhibit the extrinsic apoptotic pathway
		IRE1α/TRAF2/ASK1/Caspase-12	Inhibition↓	Alleviate endoplasmic reticulum stress-related apoptosis
Inflammatory response	Microglia	NF-κB	Inhibition↓	Decrease the transcription of pro-inflammatory cytokines
		ABIN1/A20/Cezanne	Upregulation↑	Enhance the endogenous negative regulation of NF-κB
		TLR4/MyD88	Inhibition↓	Attenuate innate immune activation
Pyroptosis	Microglia/Neurons	NLRP3/ASC/Caspase-1	Inhibition↓	Inhibit inflammasome activation
		GSDMD	Inhibition↓	Block cell membrane pore formation
		IL-1β/IL-18	Decrease↓	Reduce inflammatory amplification
		NLRP1/AIM2	Inhibition↓	Expand the regulatory scope of inflammasomes
Oxidative stress	Neurons/Astrocytes	NRF2/HO-1	Activation↑	Enhance antioxidant defense
		SOD/GSH-Px	Upregulation↑	Scavenge reactive oxygen species (ROS)
		MDA	Decrease↓	Reduce lipid peroxidation
Oxidation-inflammation coupling	Neurons/Microglia	ROS/TXNIP/NLRP3	Inhibition↓	Block the oxidative stress-inflammasome pathway
Ferroptosis	Neurons	GPX4/GSH	Upregulation↑	Inhibit lipid peroxidation
		ACSL4/TFRC	Inhibition↓	Improve iron metabolism disorders
Blood–brain barrier	Vascular endothelium	ZO-1/Occludin	Upregulation↑	Maintain tight junctions
		AQP4	Inhibition↓	Reduce cerebral edema
		METTL3/IncRNA H19/S1PR2	Inhibition↓	Protect the BBB at the epigenetic level
Angiogenesis	Neurovascular unit	HIF-1α/VEGF	Activation↑	Promote angiogenesis
		Endothelial Progenitor Cells (EPCs)/CD31	Increase↑	Improve microcirculation
		NOX2	Inhibition↓	Decrease vascular-related inflammation
Synaptic plasticity	Hippocampal neuron	BDNF/TrkB/CREB	Activation↑	Promote synaptic remodeling
		PSD-95/Synapsin-1	Upregulation↑	Enhance synaptic structural stability
		GluR2↑/NMDAR2B↓	Bidirectional modulation	Reduce excitotoxicity
		GABA-related receptors	Modulation↑	Maintain the excitation-inhibition balance
Mitochondrial homeostasis	Neurons	Mfn2/OPA1	Promotes mitochondrial fusion↑	Stabilize mitochondrial structure
		Cytochrome c	Inhibits release↓	Reduce apoptotic signals
		PGC-1α/NRF1/TFAM	Activation↑	Promote mitochondrial biogenesis
Autophagy regulation	Neurons	AMPK/mTOR/ULK1	Stage-dependent bidirectional regulation	Prevent excessive or insufficient autophagy
		PI3K/AKT/mTOR	Activation↑	Synergize energy and survival signals
		PINK1/Parkin/BNIP3L	Activation↑	Clear damaged mitochondria
Gut-brain axis	Gut-central nervous system	Short-chain fatty acids (SCFAs)/GPR43	Activation↑	Reduce gut-derived inflammation
		Treg/γδT	Immune homeostasis	Decrease systemic inflammation
		Vagus nerve	Activation↑	Neuro-immune regulation

This figure illustrates EA in the treatment of CIRI. Multiple therapeutic pathways underlie the neuroprotective effects of EA against CIRI. EA inhibits neuronal apoptosis by activating the PI3K/AKT pathway and suppressing the caspase cascade. It also mitigates inflammatory responses by reducing TLR4/MyD88 interaction, inhibiting inflammasome formation, and decreasing the release of inflammatory factors. Oxidative stress is alleviated through activation of the NRF2/HO-1 and SOD/GSHPx pathways. Additionally, EA modulates the expression of BBB-related factors, promoting protective proteins (ZO-1, Occludin) while suppressing harmful factors (AQP4, METTL3, lncRNA H19). It further attenuates ferroptosis by downregulating key components of this pathway. In terms of vascular remodeling, EA enhances angiogenesis by promoting exosome-mediated miR-210 expression. For synaptic plasticity, EA upregulates synaptic proteins such as PSD-95 and Synapsin-1, thereby improving synaptic structure and function. EA also preserves mitochondrial homeostasis by reducing cytochrome c release. Moreover, EA exerts dual regulatory effects on autophagy: during early reperfusion, it suppresses excessive autophagy via the AMPK/mTOR/ULK1 and PI3K/Akt/mTOR pathways; during the recovery phase, it promotes mitophagy through PINK1/Parkin and BNIP3L/NIX pathways to enhance mitochondrial quality control. Finally, EA further improves intestinal barrier function and modulates gut–brain immune crosstalk via microbiota–SCFA–GPR43 signaling and vagal pathways, highlighting its systemic therapeutic effects in CIRI.

## 4 Key factors influencing EA intervention in CIRI

Compared with manual acupuncture, a prominent feature of EA lies in the controllability and reproducibility of its stimulation parameters. Consequently, the therapeutic effects of EA depend not only on the pathological status of CIRI itself, but also critically on stimulation frequency, intensity, waveform, acupoint combinations, and timing of intervention. Existing studies indicate that different parameter combinations may exert distinct, or even opposing, regulatory effects on the same pathological process, which may partially account for discrepancies among reported findings. Therefore, systematic analysis of EA intervention from a parameter-oriented perspective is essential for accurate interpretation of mechanistic studies and for promoting clinical translation.

### 4.1 Characteristics and regulatory biases of EA stimulation parameters

#### 4.1.1 Selection of stimulation frequency and its biological implications

Stimulation frequency represents one of the most critical variables in the EA parameter system, as it fundamentally determines the temporal encoding of stimulation signals within the central nervous system. Current studies related to CIRI suggest that low-frequency, high-frequency, and alternating low–high frequency stimulation can all produce certain neuroprotective effects; however, their regulatory emphases differ.

Low-frequency stimulation tends to preferentially modulate neurotransmitter release and neuronal excitability, thereby contributing to improvement of neurological deficits. In contrast, high-frequency stimulation appears to show advantages in suppressing inflammatory responses and improving local cerebral blood flow. Alternating low–high frequency stimulation, commonly applied as dense–disperse waveforms, has been adopted in multiple studies and may simultaneously recruit different types of nerve fibers and central integrative pathways, resulting in a relatively balanced regulatory profile across anti-apoptotic, anti-inflammatory, and synaptic plasticity–related processes.

#### 4.1.2 Combined effects of stimulation intensity and waveform

Stimulation intensity directly affects whether EA signals can be effectively transmitted to the central nervous system and activate relevant regulatory pathways. Most studies favor moderate-intensity stimulation to achieve stable neuromodulatory effects while maintaining safety. Insufficient intensity may fail to elicit adequate central responses, whereas excessive intensity may induce stress reactions and interfere with neurological recovery.

With respect to waveform, dense–disperse stimulation, through alternating frequencies, may enhance central integration of EA signals and facilitate coordinated multi-pathway regulation. In contrast, continuous or intermittent waveforms may be effective for specific pathological indices but appear to have a more limited overall regulatory scope. Notably, low-intensity stimulation under specific acupoint conditions may activate vagus nerve–related pathways, indicating that stimulation intensity influences not only the magnitude of effects but also the recruitment of specific neuro-reflex mechanisms.

### 4.2 Acupoint strategies and patterns of integrated regulation

Acupoint selection constitutes a key component linking traditional Chinese medicine theory with modern mechanistic research in EA intervention. Different acupoint combinations exhibit distinct regulatory ranges and mechanistic emphases. Acupoint strategies represented by the “Xingnao Kaiqiao” approach emphasize modulation of central functional states through key acupoints and are characterized by broader effects on overall neurological function and widespread brain injury. In contrast, strategies represented by “Yiqi Huoxue” approaches place greater emphasis on improving cerebral microcirculation and perfusion within ischemic regions, showing advantages in angiogenesis and suppression of inflammatory responses.

In recent years, increasing attention has been directed toward combined strategies involving local acupoints and distal acupoints along meridians. This approach may improve lesion-related microenvironments through local stimulation while simultaneously modulating systemic functional states via meridian-associated and central integrative mechanisms. Such strategies reflect the principle of treating both symptoms and underlying causes and provide a potential basis for individualized EA protocols in CIRI.

### 4.3 Matching intervention timing with pathological stages

CIRI is characterized by pronounced temporal dynamics, with its predominant pathological mechanisms shifting substantially across different stages. As such, the timing of intervention emerges as a critical determinant of EA efficacy.

The ultra-early phase following reperfusion is marked by the rapid amplification of oxidative stress and inflammatory responses. EA applied during this window may be particularly effective in preventing the initiation of injury cascades by activating antioxidant pathways such as NRF2/HO-1, inhibiting inflammatory pathways such as TLR4/NF-κB, and blocking the ROS/TXNIP/NLRP3 signaling axis that couples oxidative stress and inflammation.

During the acute phase, neuronal apoptosis, sustained inflammation, and blood–brain barrier disruption predominate. EA intervention at this stage contributes to neuroprotection and containment of secondary injury, primarily through activating pro-survival pathways such as PI3K/AKT, regulating mitochondria homeostasis-related pathways, and preserving blood–brain barrier tight junctions.

As the pathological process transitions into the subacute phase, the focus gradually shifts from injury to repair. Angiogenesis, synaptic plasticity, and neural circuit reconstruction become key determinants of functional recovery. Sustained EA intervention during this period may facilitate structural and functional remodeling by activating HIF-1α/VEGF to promote angiogenesis, activating BDNF/TrkB to enhance synaptic plasticity, and modulating gut–brain axis-related pathways, thereby supporting improved long-term neurological outcomes.

Notably, the temporal evolution of CIRI pathology may also help reconcile certain findings that appear contradictory at first glance. For instance, the dual role of autophagy, where both excessive and insufficient activation can be detrimental, might reflect the need for stage-specific regulation: enhanced autophagy in the acute phase to clear damaged organelles, and restrained autophagy during repair to support cell survival. Similarly, the opposing modulation of excitotoxicity-related receptors, with GluR2 upregulation and NMDAR2B downregulation, aligns with the shifting demands of neuroprotection versus synaptic remodeling across different stages. These observations underscore that the effects of EA are not static but dynamically adapt to the evolving pathological context, reinforcing the importance of matching intervention timing with specific pathological phases.

This dynamic complexity provides a strong rationale for multi-target strategies such as EA, which can be tailored to different pathological phases through adjustments in stimulation parameters and acupoint selection to achieve integrated regulation of multiple pathways in response to stage-specific molecular events.

These observations underscore that the effects of EA are not static but dynamically adapt to the evolving pathological context, reinforcing the importance of matching intervention timing with specific pathological phases.

The therapeutic effects of EA exhibit clear parameter dependence. Key stimulation parameters, including stimulation frequency, intensity, waveform, duration, acupoint selection, and timing of intervention, may influence neural activation patterns and downstream molecular signaling pathways (Cheng N. et al., 2024). Different parameter combinations may exert distinct regulatory effects on pathological processes such as inflammatory responses, oxidative stress, neuronal apoptosis, and neurovascular remodeling (Li et al., 2025). However, despite increasing experimental evidence demonstrating the neuroprotective effects of EA in cerebral ischemia–reperfusion injury, a universally standardized EA intervention protocol has not yet been established. Considerable heterogeneity exists among current studies with respect to stimulation parameters and therapeutic strategies. Therefore, future research should prioritize systematic evaluation of parameter combinations and stage-specific intervention strategies to improve the reproducibility of findings and facilitate the clinical translation of EA therapy.

## 5 Limitations and future perspectives

Although EA regulates multiple pathological processes involved in CIRI through multi-target mechanisms, the field remains largely exploratory and lacks standardized intervention paradigms.

From an evidence hierarchy perspective, the majority of mechanistic insights are derived from preclinical animal studies. The typical experimental paradigm involves inducing cerebral ischemia–reperfusion in rodents, followed by EA intervention, with mechanistic conclusions drawn from comparing protein expression between treatment and control groups using techniques such as Western blotting. Although this approach provides evidence that EA modulates relevant pathways, it also has inherent limitations. These studies rely on static molecular measurements from whole tissue homogenates and cannot fully capture dynamic post-translational modifications, cellular specificity, or real-time signaling activity. Moreover, the translation of these findings from healthy young animal models to the complex, comorbid, and heterogeneous human patient population remains a significant challenge. Clinical studies validating these specific molecular mechanisms in humans are scarce, largely due to the invasive nature of tissue sampling. Therefore, while the evidence for EA’s interaction with these pathways in animals is substantial, the strength of evidence supporting their direct role in human neuroprotection remains indirect and should be interpreted with caution.

From a methodological perspective, substantial heterogeneity exists across studies with respect to stimulation frequency, intensity, acupoint selection, and intervention timing, limiting comparability of findings. This variability suggests that EA effects are highly dependent on parameter combinations and pathological stages; however, systematic investigations addressing the relationships among parameters, mechanisms, and therapeutic time windows remain limited.

At the mechanistic level, many studies focus on individual pathways or isolated targets. While existing evidence demonstrates that EA can modulate apoptosis, inflammation, oxidative stress, blood–brain barrier integrity, and synaptic plasticity, the temporal relationships and coordinated interactions among these pathways have not been fully elucidated. Because CIRI is highly dynamic and network-based, EA likely modulates integrated regulatory networks rather than isolated pathways.

From a translational perspective, current evidence is predominantly derived from animal studies. Mechanism-oriented clinical validation remains limited, and long-term follow-up data are often lacking, which constrains extrapolation of preclinical findings to clinical practice.

Future studies should standardize stimulation parameters, incorporate stage-specific intervention designs, and apply systems biology approaches to characterize EA-mediated regulatory networks, thereby improving interpretability and clinical relevance. To facilitate clinical translation, future studies should explore the integrated application of electroacupuncture with other traditional Chinese medicine (TCM) therapies, such as herbal medicine. Recent evidence, including the study by Liu et al. (2025), suggests that such integrative approaches may further improve therapeutic efficacy.

## 6 Conclusion

In summary, available evidence indicates that EA, through controllable stimulation parameters, exerts integrated regulation over multiple pathological processes associated with CIRI, including neuronal survival, inflammatory responses, blood–brain barrier stability, angiogenesis, and neural functional reconstruction. Rather than acting on single molecular targets, EA appears to modulate dynamic pathological regulatory networks, a characteristic that provides a theoretical basis for its application in complex brain injury. However, key research gaps remain, including limited standardization of stimulation parameters and insufficient mechanistic validation. Future research should prioritize systematic parameter optimization and multi-omics approaches to enhance reproducibility and translational relevance. Addressing these methodological priorities will be critical for advancing EA toward evidence-based clinical application in CIRI management.
